# Hemophilia A Complicated by Ulcerative Colitis

**DOI:** 10.1155/2018/2342618

**Published:** 2018-09-04

**Authors:** Maxim Olivier, Mario Madruga, S. J. Carlan, Li Ge

**Affiliations:** ^1^Department of Medicine, Orlando Regional Healthcare, Orlando, Florida, USA; ^2^Division of Academic Affairs and Research, Orlando Regional Healthcare, Orlando, Florida, USA; ^3^Department of Pathology, Orlando Regional Healthcare, Orlando, Florida, USA

## Abstract

**Background:**

Hemophilia A is an X-linked recessive disorder characterized by defective synthesis of Factor VIII protein. Depending on the level of FVIII activity, patients may present with easy bruising, inadequate clotting of traumatic or mild injury, or in severe hemophilia, spontaneous hemorrhage. Ulcerative colitis (UC) is an inflammatory bowel disease (IBD) that is reported to have a decreased frequency of occurrence in subjects with coagulation disorders.

**Case:**

A 26-year-old white male with Hemophilia A was admitted for one month of rectal bleeding. The bleeding continued despite Factor VIII replacement and colonoscopy and biopsy were performed confirming the presence of active UC.

**Conclusion:**

Ulcerative colitis with underlying F VIII deficiency can result in serious, prolonged, and possibly fatal bleeding if left unrecognized and untreated. Treatment of both conditions concurrently utilizing tertiary facilities and consultations appears to be the safest strategy for management.

## 1. Introduction

Hemophilia A is a rare and potentially lethal disease that can result in fatal hemorrhage depending on the factor activity level. It is an X-linked recessive disorder of Factor VIII (FVIII) activity and generally occurs in male children of carrier females. The diagnosis is typically made before 36 months of age and proper treatment consists of an integrated management plan including factor prophylaxis and methods to minimize bleeding risk [1]. Hemorrhage from impaired hemostasis in multiple organ systems including joints and muscle is a common clinical manifestation of Hemophilia A [2]. Occasionally the gastrointestinal tract is also involved as a bleeding site [3]. Ulcerative colitis (UC) is a chronic relapsing inflammatory intestinal condition characterized by diarrhea, cramps, and rectal bleeding, but typically not life-threatening hemorrhage [4]. Diagnosis is established by a combination of clinical, radiographic, endoscopic, and histologic work-up. Thrombosis and vascular occlusion may be important in the pathogenesis of US and disorders of coagulation may be protective against UC [5]. Consequently, the combination of Hemophilia A and UC is extremely unusual. We present a case of a young male with hemophilia A who presented for rectal bleeding and was newly diagnosed with ulcerative colitis.

## 2. Case

A 26-year-old white male presented to the emergency department complaining of one month of persistent hematochezia, lower abdominal pain, and nonbloody emesis. He noted intermittent rectal bleeding for years but never continuously for 1 month. He denied any fevers, weight changes, sick contacts, antibiotic use, or previous colonoscopy. His past medical history included moderate Hemophilia A with factor activity between 1 and 5 percent. He had been prescribed Recombinant human factor VIII but had been noncompliant with visits. When his rectal bleeding increased he was told to take his Recombinant human factor VIII to 4000 units every 12 hours on days that he has heavy bleeding.

His examination was significant for tachycardia, tenderness to palpation to the lower abdomen, and positive fecal occult blood testing. Serum laboratory testing revealed a hemoglobin of 11 g/dL and PT/PTT/INR values within normal limits. His C reactive protein was elevated at 57.8 mg/L (normal ≤ 9.9), erythrocyte sedimentation rate 21 mm/hr (normal 0-15), and albumin 3.5 g/dL (normal 3.5-5.7).

**Figure 1:**
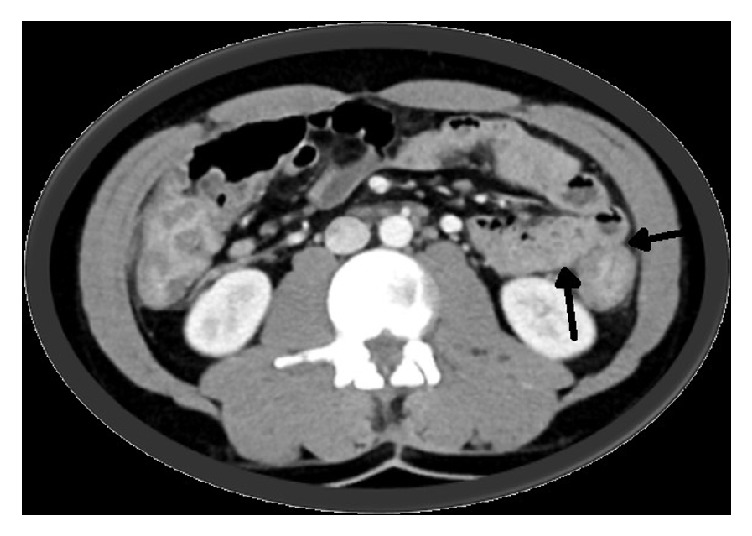
Transverse abdomen CT. Diffuse abnormal colonic thickening consistent with pancolitis (arrows). The small bowel is normal.

**Figure 2:**
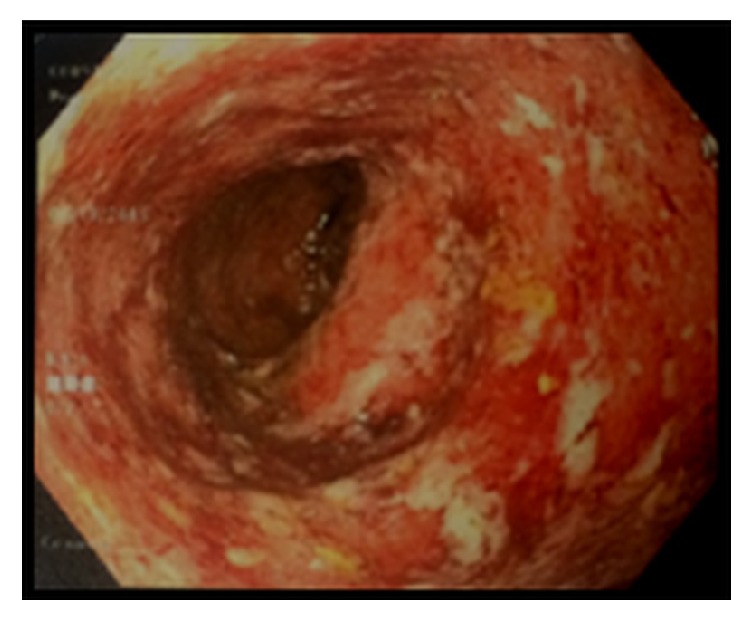
Sigmoid colon at colonoscopy. Inflammation characterized by adherent blood, congestion (edema), erosions, erythema, friability, granularity, and mucus was found in a continuous and circumferential pattern.

**Figure 3:**
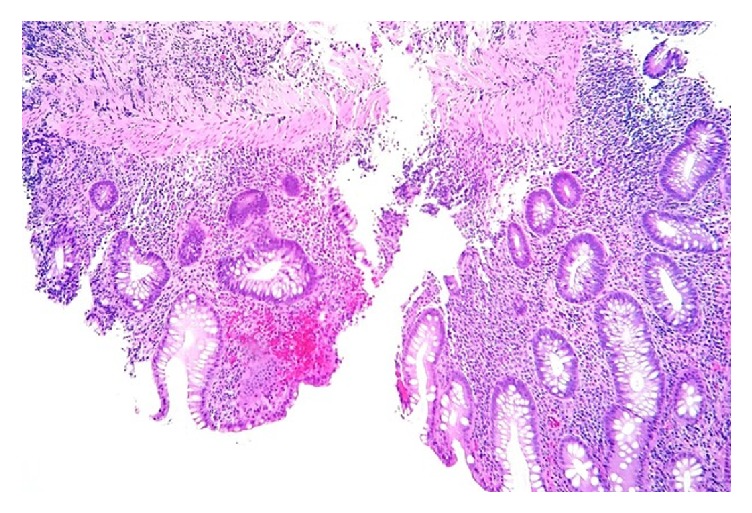
Biopsy of rectum shows chronic active colitis; histopathology and anatomic distribution of disease are consistent with ulcerative colitis (hematoxylin & eosin stain; original magnification x100).

CT imaging of the abdomen and pelvis revealed diffuse abnormal colonic thickening (Figure 1). After receiving Recombinant human factor VIII infusions without resolution of symptoms, a decision was made to perform colonoscopy (Figure 2) and biopsies which confirmed chronic active ulcerative colitis (Figure 3). He was started on empiric therapy for inflammatory bowel disease and eventually required high-dose intravenous steroids before he showed clinical improvement. Intravenous methyl prednisolone 40 mg every six hours was given for the first six days followed by 60 mg daily intravenously until discharge. He was given budesonide 9 mg orally once daily for the first six days, mesalamine 800 mg orally three times a day for the first six days, and pantoprazole 40 mg orally for his entire 14-day inpatient stay. He also received metronidazole 500 mg intravenously once daily for the first six days and then orally every eight hours until discharge. Stool cultures showed the usual enteric flora and HIV was negative. His hospitalization was complicated by blood loss anemia, requiring multiple blood transfusions in addition to twice daily dosing of Recombinant human factor VIII. He was eventually discharged after 14 days on an oral steroid taper and, as an outpatient, was to be followed up for initiation of a biologic agent. After discharge the patient was lost to follow-up.

## 3. Discussion

This case is unique for several reasons. First, venous thrombosis appears to be increased in patients with inflammatory bowel disease suggesting that Hemophilia A may provide a protective effect [5]. In fact, there is a significantly lower prevalence of ulcerative colitis in patients with hemophilia making this case even more unusual [6]. The reasons for this are only speculative but probably reflect a local bowel thrombogenic characteristic in patients who develop UC. Second, therapeutic options are initially undefined because the etiology of the bleed could represent a straightforward expression of FVIII deficiency or the UC alone despite data showing less than 4% of ulcerative colitis patients suffer massive hemorrhage [4]. Nonetheless, immediate control of hemorrhaging is critical and in our case the subject continued to bleed despite Recombinant human factor VIII infusions. This suggested a more complex disease spectrum which was confirmed with the colonoscopy and biopsy proven ulcerative colitis. Third, data on concurrent Hemophilia A and ulcerative colitis are virtually nonexistent other than several case reports and opinions. Consequently, there are no evidence-based protocols for treating or preventing further episodes other than what is recommended for each condition separately.

This case illustrates that comorbid UC with underlying F VIII deficiency can result in serious, prolonged, and possibly fatal bleeding if left unrecognized and untreated. Treatment of both conditions concurrently utilizing tertiary facilities and consultations appears to be the safest strategy for management. Prevention of UC flares and bleeding episodes may also require sophisticated follow-through health monitoring which includes counseling for patient compliance struggles.
